# Optimization of culture conditions for short-term maintenance, proliferation, and colony formation of porcine gonocytes

**DOI:** 10.1186/s40104-017-0222-0

**Published:** 2018-01-17

**Authors:** Awang Hazmi Awang-Junaidi, Ali Honaramooz

**Affiliations:** Department of Veterinary Biomedical Sciences, Western College of Veterinary Medicine, University of Saskatchewan, 52 Campus Drive, Saskatoon, SK S7N 5B4 Canada

**Keywords:** Cell culture, Male Germline stem cells, Pigs, Testis

## Abstract

**Background:**

Gonocytes give rise to spermatogonial stem cells, and thereby play an essential role in establishing spermatogenesis. Optimized culture conditions for gonocytes provide an opportunity for their study and in vitro manipulation for potential application in reproductive technologies. Using six experiments in a step-wise design, we examined the effects of several culture conditions on the maintenance, proliferation, and colony formation of porcine gonocytes. Testis cells from neonatal piglets were cultured for 7 d in DMEM supplemented with 10% fetal bovine serum. The examined culture conditions included using different cell seeding densities, gonocyte proportions, incubation temperatures, sampling strategies, and medium changing regimens.

**Results:**

Confluency of cells was optimal (>90% by ~6 d) when 3.0 × 10^4^ testis cells/cm^2^ containing ~40% gonocytes were used. Incubating the cells at 35 °C or 37 °C resulted in similar cell number and viability at confluency, but incubation at 35 °C resulted in a delayed confluency. In the first 2 d of culture, gonocytes remained mostly floating in the medium and gradually settled over the next 5 d. Consequently, not changing the medium for 7 d (as opposed to changing it every 2 d) led to a significant increase in the number of gonocyte colonies by reducing the loss of “floating gonocytes”.

**Conclusion:**

We found that gonocytes require the presence of a critical minimum number of somatic cells for settlement, and can proliferate and form growing colonies even in a basic medium. Large numbers of viable gonocytes remain floating in the medium for several days. The optimized culture conditions in the present study included seeding with 3.0 × 10^4^ testis cells/cm^2^ containing ~40% gonocytes, incubating at 37 °C, and without changing the medium in the first week, which can result in improved colony formation of porcine gonocytes.

## Background

Testis stem cells are a unique population of stem cells in an adult body, because in addition to their dual ability to self-renew and give rise to differentiating germ cells, they can pass the genetic information to the offspring. Among the many types of male germ cells present from the fetal period to adulthood, primordial germ cells (PGCs), gonocytes, and particularly spermatogonial stem cells (SSCs) are believed to possess stem cell potential and hence are referred to as male germline stem cells (MGSCs) [1]. An in vivo functional assay for confirming the stem cell potential of MGSCs was provided when SSCs were deposited in the lumen of seminiferous tubules of recipient testes using germ cell transplantation technique. The transplanted SSCs were capable of homing to the tubule basement membrane, forming new colonies of developing germ cells, and initiating donor-derived spermatogenesis [2–7]. In breeding trials, some of the germ cell recipient animals of various species were capable of siring donor-derived progeny [2, 6, 8]. There are also a few reports of using gonocytes and PGCs as donor cells in germ cell transplantation leading to the establishment of spermatogenesis in the recipient testis [3, 9, 10].

The choice of donor MGSCs is a critical factor for the outcome of germ cell transplantation. Using PGCs as donor cells has been less practical because their isolation requires collection from embryos and there are only a limited number of PGCs per embryo [3, 11]. Spermatogonia can be collected from mature donor individuals and enriched for SSCs at relatively high numbers. However, spermatogonia are inherently a heterogeneous population of cells and the lack of biomarkers that allow unequivocal isolation of SSCs has hampered obtaining pure populations [12–14]. Compared with PGCs and SSCs, gonocytes offer a potentially more practical option. Gonocytes are transitory germ cells (between PGCs and SSCs) that have a distinct morphology (large round cells with large nucleus to cytoplasm ratio), and are present during both fetal and early postnatal period for several days, months or even years, depending on the species [15–17]. Perhaps more importantly, gonocytes are the only germ cells present in the testes of neonates; thus, they can be detected using most germ cell markers (e.g., VASA, DAZL), pluripotency markers (e.g., NANOG, OCT4), and certain surface protein markers (e.g., DBA, UCH-L1). These characteristics provide an opportunity for enrichment and in vitro manipulation of gonocytes [17–20].

Gonocytes are relatively limited in numbers since they comprise only ~1.4% of cells in the testes of neonatal rats [21] and ~7% of seminiferous cord cells in piglets [22]. The efficiency of colonization by the donor germ cells in the seminiferous tubules of recipients is directly proportional to the number of transplanted MGSCs. Therefore, to increase the efficiency of germ cell transplantation, gonocytes must be enriched and/or propagated in vitro prior to transplantation, as suggested for SSCs [23, 24]. Various strategies have been introduced to optimize the isolation, enrichment, or purification of gonocytes with differing outcomes, depending on the species [18, 25, 26]. Using conventional cell separation methods, no more than ~10% of isolated neonatal testis cells were gonocytes [21, 22, 27, 28]. However, development of a three-step enzymatic digestion in our laboratory has allowed the collection of populations of cells containing ~40% gonocytes from neonatal porcine testes [29]. The resultant population of gonocytes can also be enriched further to ~90% using a combination of Nycodenz centrifugation and differential plating [30].

While pigs are an important biomedical animal model, purification of porcine MGSCs is still limited. The ability to obtain highly enriched populations of gonocytes from neonatal piglets [29, 30], however, has provided new opportunities for using gonocytes as a model for in vitro propagation and manipulation of MGSCs. This calls for further research on culture systems that can allow efficient large-scale propagation of gonocytes. No systematic study has examined the effects of different culture conditions on colony formation of porcine gonocytes. Therefore, given the importance of cell culture in MGSC research, the present study was designed to examine a number of culture conditions for maintenance, proliferation, and colony formation of neonatal porcine gonocytes as a model. In the present study, we performed six experiments in a stepwise design and examined several aspects of testis cell culture including factors related to the seeding density, gonocyte proportion, incubation conditions, sampling, medium changing regimens, and effects of applying those optimal conditions on gonocyte colony formation.

## Methods

### Testis collection and preparation

Testes from Yorkshire-cross piglets (Camborough-22 × Line 65; PIC Canada, Winnipeg, MB, Canada) less than 1 wk. of age were used in this study. The testes were collected weekly (5–15 pairs per week) through aseptic castration at a university–affiliated swine facility. The testes were kept in ice-cold Dulbecco’s phosphate-buffered saline (DPBS; catalogue no. 20–031-CV; Mediatech, Manassas, VA, USA), containing 1% w/v antibiotics solution (penicillin and streptomycin; catalogue no. 30–002-CI, Mediatech), and were transported within 1 h of collection to the laboratory. The testes were thoroughly rinsed three times with DPBS prior to processing. The parenchyma was separated from the tunica albuginea and excess connective tissues, and used for isolation of testis cells.

### Isolation of testis cells and enrichment for gonocytes

Isolation of testis cells and enrichment of gonocytes were performed using methods that were established in our laboratory (three-step enzymatic digestion, Nycodenz density gradient centrifugation and extracellular matrix (ECM) differential plating; [29, 30]). Briefly, for each batch of the three-step enzymatic digestion, ~600 mg of testis parenchyma was thoroughly minced with fine scissors for 5 min, suspended in 5 mL of DPBS, vortexed for 30 s in a test tube shaker (Reax Top; catalogue no. 541–10000; Heidolph Instrument, Essex, UK) and digested with 1 mL of 0.2% w/v collagenase IV (catalogue no. C-153; Sigma-Aldrich, Oakville, ON, Canada), 0.1% w/v hyaluronidase (catalogue no. H-3884; Sigma-Aldrich), and 0.01% DNase (catalogue no. DN25; Sigma-Aldrich) in Dulbecco’s modified Eagle’s medium (DMEM; catalogue no. 10–013-CM; Mediatech) supplemented with 1% w/v antibiotics (as above) at 37 °C for 10 min. Fetal bovine serum (FBS; catalogue no. A15–701; PAA Laboratories, Etobicoke, ON, Canada) was added to stop the digestion and the suspension was vortexed for another 30 s and filtered through a 40-μm filter (catalogue no. 3522340; BD Biosciences, San Jose, CA, USA). The filtrate suspension was centrifuged at 500×*g* at 16 °C for 5 min and resuspended in 5 mL of DPBS supplemented with 1% w/v antibiotics. Erythrocyte depletion was performed by mixing the cells with 20 mL of the lysis buffer, composed of 156 mmol/L ammonium chloride (NH_4_Cl; catalogue no. A9434; Sigma-Aldrich), 10 mmol/L potassium bicarbonate (KHCO_3_; catalogue no. 237205; Sigma-Aldrich), and 0.1 mmol/L disodium ethylenediaminetetraacetate (Na_2_EDTA; catalogue no. E6635; Sigma-Aldrich) in sterile distilled water prior to another cycle of centrifugation (500×*g* at 16 °C for 5 min). Finally, the cell pellet was resuspended in 5 mL of DMEM supplemented with 10% v/v FBS and 1% antibiotics (DMEM^++^) and underwent gentle pipetting to obtain a single cell suspension.

For enrichment of gonocytes among the cells obtained from the three-step enzymatic digestion, 3 mL of 17% Nycodenz in DPBS (Histodenz; catalogue no. D2158; Sigma-Aldrich) was placed at the bottom of a 15-mL graduated conical tube. This was followed by gentle addition of 2 mL of cell suspension on top and the tube was centrifuged at 500×*g* at 4 °C for 15 min. The supernatant was discarded and the pellet was harvested and resuspended as a single cell suspension. Prior to ECM differential plating, 6-well culture plates (catalogue no. 353046; BD Biosciences) were coated with 1 mL of 50 μg/mL poly-D-lysine (catalogue no. 47743–736; VWR International, Mississauga, ON, Canada) and 10 μg/mL fibronectin (catalogue no. 477743–728; VWR) in an incubator with 5% CO_2_ at 37 °C for 1 h, and dried in a biosafety cabinet for another 1 h. The poly-D-lysine pre-coated wells were rinsed twice with DPBS before seeding the cells obtained from Nycodenz gradient density centrifugation. The cells were seeded onto the plates at a concentration of 2.5 × 10^5^ cells/cm^2^ in DMEM^++^, and cultured in an incubator with 5% CO_2_ at 37 °C. After 2 to 3 h, the floating cells were harvested, centrifuged at 500×*g* at 16 °C for 5 min and the pellets were collected and resuspended as a single cell suspension. These procedures lead to obtaining isolated testis cells highly enriched in gonocytes. We performed a side-by-side quantitative comparison of the cell yield, viability and loss after each progressive step. Samples were prepared from single cell suspensions obtained from each step. The suspension was smeared onto poly-L-lysine pre-coated glass coverslips, dried overnight in a biosafety cabinet and kept at −20 °C for use in immunostaining.

### Cell quantification and viability assessment

The number of resultant cells after each isolation and enrichment step as well as after culturing was quantified, and the cell viability was assessed using the trypan blue exclusion technique. For evaluation of cells collected after isolation and enrichment, single cell suspensions were prepared as described above. For cultured cells, detachment of the adherent cells was performed by adding 1 mL of 0.25% (w/v) trypsin in Hank’s balanced salt solution (HBSS) and 2.21 mmol/L EDTA (catalogue no. 25–053-CI; Mediatech) at 37 °C for 1–2 min (depending on confluency) with gentle agitation. The enzymatic digestion was stopped by adding 1 mL of undiluted FBS, and the mixture was centrifuged at 500×*g* at 16 °C for 5 min. The pellets were collected and subjected to resuspension into a single cell suspension prior to assessment. Trypan blue (100 μL of a 0.4% solution in saline, catalogue no. T8154; Sigma-Aldrich) was mixed (1:1 ratio) gently with the cell suspension, and 20 μL of the mixture was transferred to each hemocytometer chamber and live/dead cells were identified with the aid of a light microscope. For each replicate, two counts of live/dead cells were performed and averaged to calculate the yield and viability of cells (given as a percentage). Immunostaining was performed to confirm and quantify gonocytes, as they are the only type of germ cells present in neonatal pig testes; hence the remaining cells were categorized as somatic cells. For immunostaining, a minimum of 600 cells in randomly selected fields were counted in each cell smear and the proportion (%) of gonocytes/somatic cells was calculated.

### Cell culture

Different cell culture conditions were evaluated using a stepwise approach. The same medium (DMEM^++^), culture plates (6-well plates), and incubator conditions (5% CO_2_ and 95% humidity) were used in all experiments in order to evaluate the effects of changing other variables (i.e., gonocyte proportion, incubation temperature, cell seeding density, and medium changing regimen). For all experiments, pH of the medium was adjusted to 7.4 immediately prior to using it in cell cultures. The pH of the medium was measured using a digital pH meter (catalogue no. 11288–368; model Symphony SB70P; VWR). Cell growth was monitored at least once daily using an inverted phase contrast microscope (Nikon, Eclipse TS100). For each experiment, multiple replications were performed where cells obtained from each batch of testis tissue digestion were considered one replication. We have previously shown that the characteristics of cells obtained from 1-week old piglets from this source remain consistent over time, both within and among litters [29].

Effects of several factors on the behavior and the morphology of cultured testis cells were examined using cytology and immunostaining. The examined factors included different gonocyte proportions obtained before or after each step of enrichment (40% vs. 80% vs. 90%), incubation temperature (35 °C vs. 37 °C), cell seeding density (1.0 × 10^5^ vs. 3.0 × 10^5^ vs. 5.0 × 10^5^ cells/well; each well in a 6-well plate has ~10 cm^2^ cell growth area) and medium changing regimen (every 2 d vs. modified regimen vs. no changing of medium for up to 7 d). Confluency assessment was defined as the relative coverage of the plate surface by the mixture of testis cells, and a gonocyte colony was defined as a group of more than three gonocytes in close contact.

### Sampling

For sampling, glass coverslips coated with 0.1% w/v poly-L-lysine were used. Glass coverslips sized 22 × 22 mm (catalogue no. 12-540B; Fisher Scientific, USA) were arranged in a sterile metal coverslip rack and immersed in 1% HCl in 70% alcohol for 10 min, followed by rinsing with sterile distilled water for 5 s and allowed to dry for 20 min. The rack was transferred into a 0.1% w/v poly-L-lysine (catalogue no. P8920; Sigma-Aldrich) solution for 10 min and allowed to dry overnight. Finally, the glass coverslips were placed at the bottom of each well in 6-well plates. These procedures were conducted under a biosafety cabinet.

### Immunostaining

**Fig. 1:**
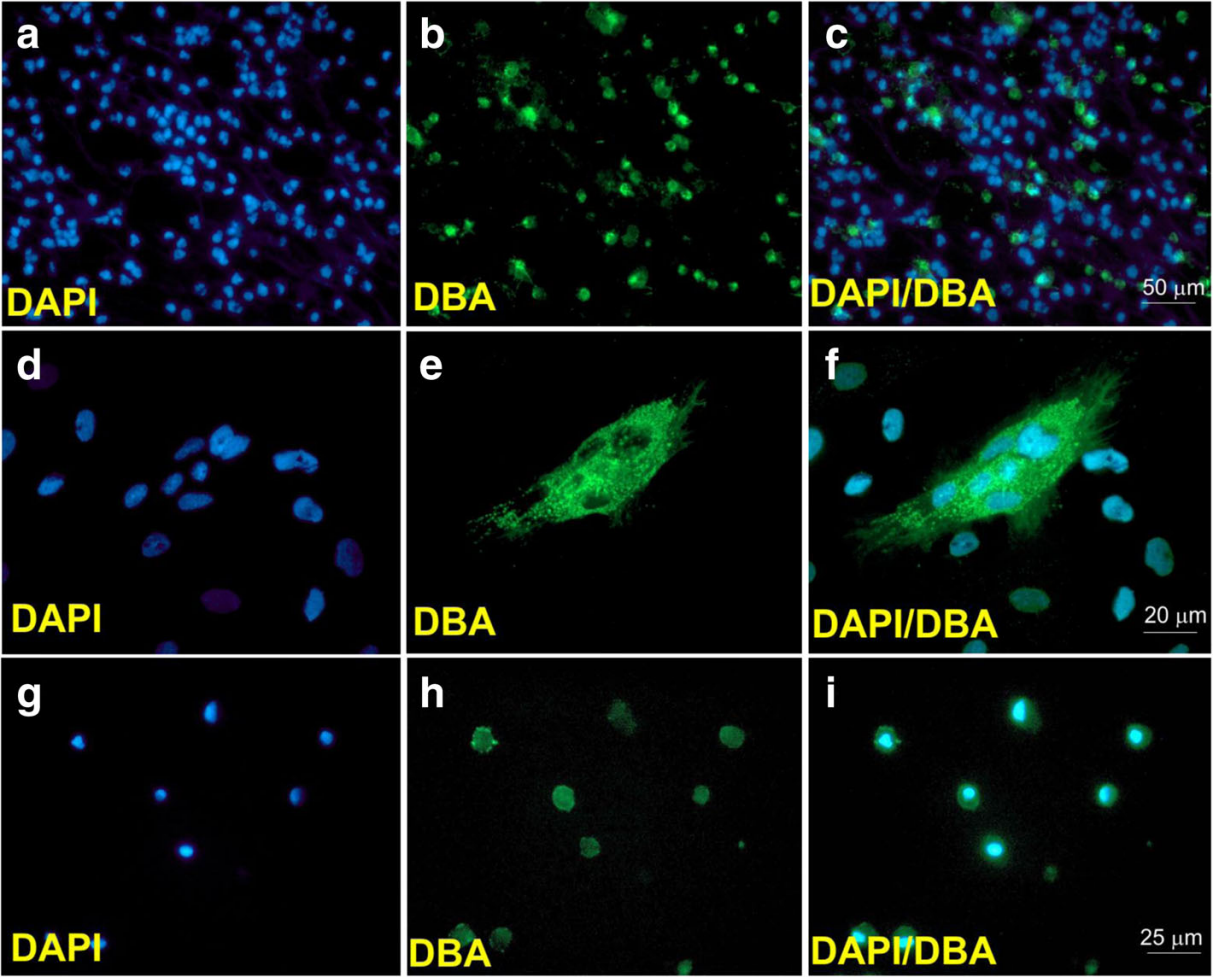
*Dolichos biflorus* agglutinin (DBA) immunostaining of neonatal gonocytes. **a**–**c** Gonocyte immunostaining performed on testis cell smears collected immediately following cell isolation. **d**–**f** Multiple gonocytes form a colony on a somatic-cell monolayer observed at 1 wk of culture. **g**–**i** Floating gonocytes in the removed medium observed 4 d after incubation. **b**, **e**, **h** Fluorescein-conjugated DBA-labeled gonocytes appear green under the fluorescence microscope. **a**, **d**, **g** DAPI-stained nuclei of the cells appear blue. **c**, **f**, **i** Merged images

Gonocytes were identified by immunostaining using *Dolichos biflorus* agglutinin (DBA) according to previously described protocols [29, 30], with minor modifications. Briefly, the dried coverslips (with cell smears or with cells grown directly on them) were fixed in Bouin’s solution (catalogue no. 1120–31; Richa Chemical Company, Pocomoke City, MD, USA) for 2–3 min. The samples were then rinsed three times with 70% alcohol to remove excess Bouin’s solution followed by three rinses with DPBS. The samples were blocked with 5% bovine serum albumin (BSA; catalogue no. 0332, Amresco, Solon, OH, USA) at 37 °C for 30 min in a humidified chamber. The blocking agent was removed and the samples were incubated with fluorescein-conjugated lectin DBA (1:100, catalogue no. FL-1031, Vector Labs, Burlington, ON, Canada) overnight at 4 °C in a humidified chamber. The samples underwent another cycle of rinsing, followed by incubation with 0.3% Sudan Black B w/v (catalogue no. S2380; Sigma-Aldrich) in 70% alcohol for 15 min, at 37 °C in a humidified chamber. The samples were rinsed with DPBS and stained with 4′6’-diamidino-2-phenyilindole (DAPI; catalogue no. D9542; Sigma-Aldrich) and observed under a fluorescence microscope. Fluorescein-conjugated lectin DBA specifically labels gonocytes which appear green under the fluorescence microscope while DAPI stains the nuclei of all cells which appear blue (Fig. 1).

### Statistical analyses

Unless stated otherwise, all data are presented as means ± standard error of mean (SEM) and analyzed using either one-way or two-way analysis of variance (ANOVA), as appropriate, followed by Tukey’s HSD post-hoc test. For percentages, the data were transformed using Arcsine function prior to analysis using ANOVA. The level of significance was set at *P* < 0.05. Data were analyzed using SPSS (Version 20.0; SPSS, Chicago, IL, USA).

## Results

As summarized in Table 1, enzymatic digestion of testis tissues yielded a population of testis cells containing ~41% germ cells (Fig. 1a-c), which at this developmental stage (~1 wk of age) are exclusively gonocytes. Gradient centrifugation by Nycodenz, followed by ECM differential plating resulted in enrichment of gonocytes to ~80% and ~86%, respectively. However, the enrichment procedures also caused significant reductions (*P* < 0.05) in cell counts after each step, resulting in ~73% and ~92% total testis cell loss and ~47% and ~83% gonocyte loss, respectively. The relatively lower gonocyte loss at each step, compared with the total cell loss, resulted in significant enrichment of gonocytes. The viability of cells was also reduced after each enrichment step (from ~93% to 84% and 82%, respectively, *P* < 0.05). When the cells were cultured (3.0 × 10^5^ cells/well at 37 °C with 5% CO_2_ and the medium was changed every 2 d), only the cells used immediately after the three-step enzymatic digestion (i.e., without enrichment) demonstrated growth by reaching ~90% confluency in ~6 d.

**Table 1:** The population of testis cells obtained after enzymatic digestion and subsequent enrichment

Method	Proportion, %		Cell count, × 10^6^ *		Cell loss, % **		Viability, %	In vitro growth
	Gonocytes	Somatic cells	Total	Gonocytes	Total	Gonocytes		
Enzymatic digestion	40.60 ± 1.21 ^a^	59.40 ± 1.21 ^a^	37.84 ± 2.44 ^a^	15.38 ± 1.09 ^a^	–	–	92.62 ± 0.59 ^a^	Yes
Nycodenz centrifugation	80.40 ± 1.46 ^b^	19.6 0 ± 1.46 ^b^	10.00 ± 0.41^b^	8.00 ± 0.21 ^b^	73.18 ± 1.76 ^a^	46.72 ± 4.69 ^a^	84.20 ± 1.06 ^b^	Limited
ECM differential plating	86.40 ± 1.03 ^c^	13.6 0 ± 1.03 ^c^	3.02 ± 0.19 ^c^	2.60 ± 0.2 ^c^	91.62 ± 0.79 ^b^	82.61 ± 1.71 ^b^	81.66 ± 0.71 ^c^	Limited

The results are mean ± SEM

*Each replicate was based on the digestion of ~600 mg of testis tissue

One-way ANOVA (or *t* test **) was used for statistical analyses, and *P* < 0.05 was considered as significant

^abc^Within each column, data with different superscripts differ significantly (*P* < 0.05). *n* = 5 replications

### Exp. 1: Effect of cell seeding density and gonocyte proportion

One of the objectives of this experiment was to determine the optimal seeding density for culturing testis cells. A comparison was made by randomly assigning cells into 6-well plates at 1.0 × 10^5^, 3.0 × 10^5^, or 5.0 × 10^5^ cells/well (i.e., 1.0 × 10^4^, 3.0 × 10^4^, or 5.0 × 10^4^ cells/cm^2^ since each well was ~10 cm^2^, *n* = 12 replications), in 3 mL of medium to determine the time required for the cells to reach 90% confluency. Cells were used fresh (immediately after enzymatic digestion and without enrichment, hence containing ~40% gonocytes) and cultured under similar conditions at 37 °C, and the medium was changed every 2 d.

As shown in Table 2, cells in the highest seeding density group (5.0 × 10^5^) became confluent earlier than other groups (*P* < 0.05), reaching >90% confluency in ~4 d, while the 3.0 × 10^5^ seeding density group of cells were >90% confluent by ~Day 6. Cells cultured at 1.0 × 10^5^ seeding density had slow growth and only reached ~30% confluency after 7 d in culture.

**Table 2:** The effect of seeding density on the growth of testis cells in culture

Cell seeding density	Maximum confluency		
	Confluency at days	Cell count	Viability, %
1.0 × 10^5^	(*~30% at 7)	(*2.98 × 10^5^ ± 0.01)	(*88.4 ± 1.02)
3.0 × 10^5^	>90% at 6.0 ± 0.17 ^a^	8.10 × 10^5^ ± 0.01	91.21 ± 0.58
5.0 × 10^6^	>90% at 4.0 ± 0.15 ^b^	8.16 × 10^5^ ± 0.01	91.62 ± 0.56

The results are mean ± SEM

*The results from 1.0 × 10^5^ seeding density group were excluded from statistical analysis as the cells did not reach 90% confluency

Independent sample *t* test was used for statistical analyses

^ab^Within each column, data with different superscripts differ significantly (*P* < 0.05). *n* = 12 replications

In a separate experiment, testis cells enriched for gonocytes (undergoing both Nycodenz centrifugation and ECM plating, hence containing ~86% gonocytes) were used as the source of cells for culturing, using the same incubation conditions. Cells did not grow in plates seeded with 1.0 × 10^5^ or 3.0 × 10^5^ cells/well and only had limited growth in the 5.0 × 10^5^ seeding density group after 7 d in culture (5–15% confluency, data not shown). Therefore, for the remaining experiments in this study we used testis cells without enrichment (i.e., containing ~40% gonocytes), and since the 3.0 × 10^5^ seeding density resulted in confluency of cells in ~6 d (without the need for passaging the cells within 1 wk), we used this seeding density in the subsequent experiments.

### Exp. 2: Effect of incubation temperature

The objective of this experiment was to evaluate the growth of cultured testis cells using incubation temperatures of either 35 °C or 37 °C for 1 wk (*n* = 12 replications), while other culture conditions remained the same (e.g., medium, cell seeding density, and CO_2_ levels). As shown in Table 3, the viability and number of cells at confluency did not differ between the two incubation temperatures (*P* > 0.05). However, the cells cultured at 35 °C reached confluency later than those at 37 °C (*P* < 0.05); therefore, for practical reasons we chose to culture the cells at 37 °C for subsequent experiments.

**Table 3:** The effect of incubation temperature on the growth of testis cells in culture

Temperature	>90% confluency		
	Days	Cell count	Viability, %
35 °C	6.42 ± 0.15 ^a^	7.98 × 10^5^ ± 0.01	89.98 ± 0.49
37 °C	5.83 ± 0.17 ^b^	8.27 × 10^5^ ± 0.01	90.04 ± 0.49

The results are mean ± SEM

Independent sample *t* test was used for statistical analyses

^ab^Within each column, data with different superscripts differ significantly (*P* < 0.05). *n* = 12 replications

### Exp. 3: Sampling of cultured testis cells for analysis

**Fig. 2:**
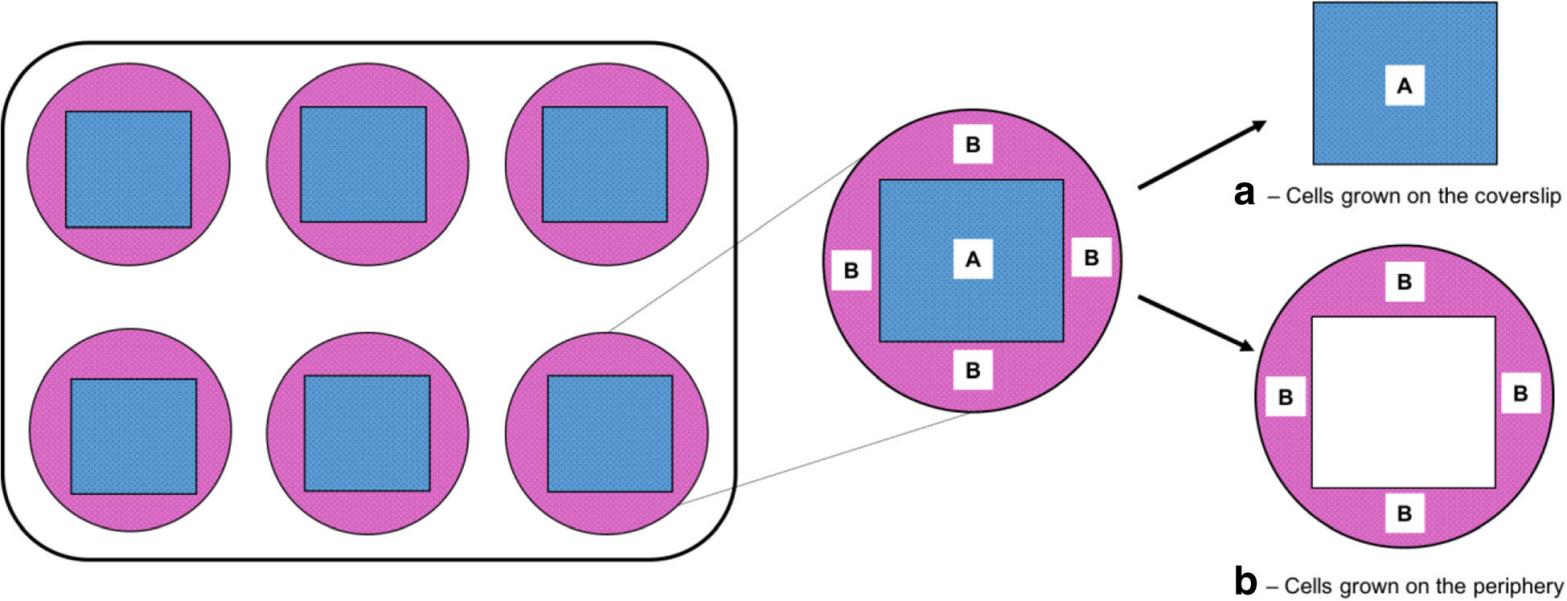
Schematic representation of the areas within culture plates used for sampling in Exp. 3. The numbers and viability of cells grown on poly-L-lysine-coated coverslips (**a**) were compared with those grown on the peripheral areas of the wells, not covered by the coverslip (**b**)

Various methods can be used to sample cultured cells for analysis. When sampling for applications such as immunocytochemistry, coverslips are commonly pre-coated with certain matrices to enhance adherence of cells. It is not clear whether the presence of the pre-coated coverslips in testis cell culture would affect cell growth. Furthermore, in situations where multiple samplings from each replicate is required, it would be more efficient to use all available cultured cells, including those grown on areas of the culture well not covered by the coverslip. The objectives of this experiment were to determine 1) whether the rate or pattern of testis cell growth in culture would change in the presence of glass coverslips (22 × 22 mm) coated with 0.01% poly-L-lysine, and 2) whether the growth of testis cells on the coverslips differs from those on the peripheral areas of the wells, not covered by the coverslips (Fig. 2).

When plates with and without poly-L-lysine-coated coverslips were compared, the number or viability of the resultant cultured cells did not differ between plates (8.18 ± 0.01 × 10^5^ vs. 8.22 ± 0.02 × 10^5^ cells per plate, with 91.0 ± 0.57% vs. 91.3 ± 0.87% cell viability, plates with coverslips vs. plates with no coverslips, respectively, *n* = 12 replications, *P* > 0.05). When non-coated coverslips were used, little or no cell growth was observed. Furthermore, within the plates with poly-L-lysine-coated coverslips, the viability of cells grown on coverslips did not differ from those grown on the peripheral areas of the well, not covered by the coverslips (92.0 ± 0.33% vs. 92.4 ± 0.51%, coverslips vs. periphery, respectively, *P* > 0.05). However, the number of cells obtained from the peripheral areas was higher than that from the coverslips (4.59 ± 0.14 × 10^5^ vs. 2.99 ± 0.06 × 10^5^, respectively, *P* < 0.05), even though both encompassed a similar cell growth area (~4.8 cm^2^ each).

### Exp. 4: Short-term culture of neonatal porcine testis cells

**Fig. 3:**
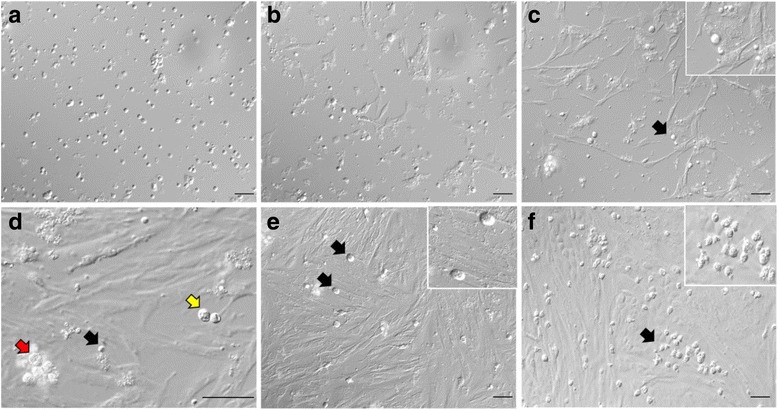
Morphology of testis cells in short-term cell culture. **a** Morphology of floating cells 12 h after seeding. **b** Some somatic cells settled to the bottom of the plate within 24 h of incubation. **c** These somatic cells extended cytoplasmic projections toward one another. **c**–**f** Cells presumed to be gonocytes are seen attached to somatic cells (black arrows). The same gonocytes are also shown at a higher magnification (insets). **d** Gonocytes appeared as singles (black arrow), pairs (yellow arrow), or small colonies (red arrow). **e** Spindle-shaped fibroblast-like cells were more prominent when confluency reached >70%. Only a small number of gonocyte colonies were observed by Day 7 when routine medium changing regimen was applied. **f** Increased number of gonocyte colonies were observed when the medium was not changed for 7 d. Scale bar, 50 μm

Morphological changes of neonatal porcine testis cells in culture were evaluated for 1 wk under the above-mentioned culture conditions (3.0 × 10^5^ seeding density, DMEM^++^, 37 °C, *n* = 6 replications). The medium was changed every 2 d. Within the first 12 h of incubation, most of the cells and cell particles were still floating in the medium (Fig. 3a). By 24 h after incubation, small numbers of cells settled and appeared to have adhered to the plate. The early settling cells were mostly somatic cells and began to form cytoplasmic projections, creating irregularly shaped cell boundaries (Fig. 3b), while more cells continued to settle. After 2 d of incubation, the cells started to appear in clumps (Fig. 3c), and over time these groups of cells spread further and combined to create a large somatic-cell monolayer. At the same time, round cells with large nuclei, appearing bright or with a halo under the phase-contrast microscopy, were seen attached onto the somatic-cell monolayer (Fig. 3d–e). These rounds cells were confirmed to be gonocytes using immunostaining (Fig. 1d–f). It was also observed that while initially gonocytes were present as singles or pairs, starting at Day 5, colonies (grape-like clumps) of more than 3 cells could also be observed (Fig. 3d). It was noticed that gonocytes were only loosely attached to the somatic-cell monolayer, since some would detach due to slight movements of the plate. By the end of the experiment (Day 7), the number of these round cell colonies were still considerably less than expected, given that the population of testis cells at seeding contained ~41% gonocytes. The somatic cells continued to multiply and once reaching 70–80% confluency (at ~4 to 5 d), many appeared as spindle-shaped fibroblast-like cells (Fig. 3e).

### Exp. 5: Floating cells in the culture medium

**Fig. 4:**
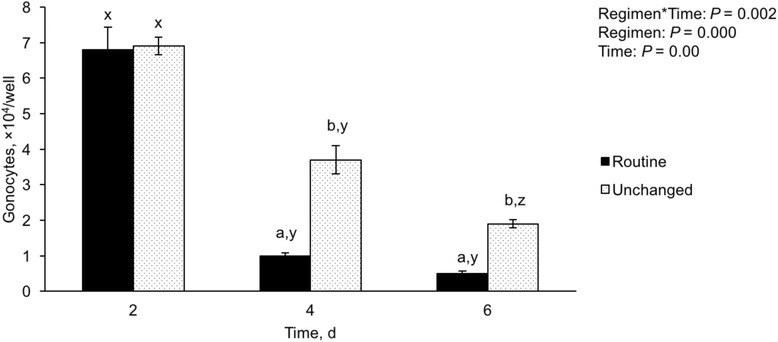
Number of gonocytes upon analysis of the floating cells from the removed medium. The initial number of cells at seeding was set at 3.0 × 10^5^ cells/well containing ~40% gonocytes and the viability was 89 ± 0.5%. The medium removed from the plates was examined and large numbers of gonocytes were seen floating in the medium on Day 2. Most of the floating gonocytes were eliminated by routine (every 2 d) medium changes (Routine), while some were present on Days 4 and 6, if the medium was not changed (Unchanged). The data are mean ± SEM. ^ab^ Data with different superscripts within each day differ significantly (*P* < 0.05). ^xyz^ Data with different superscripts within each treatment over time differ significantly (*P* < 0.05). *n* = 5 replications

Based on the findings of Exps. 1 and 4, it was clear that gonocytes require sufficient ratios of somatic cells or feeder cells, to which they formed loose attachment for growth in culture. We speculated that the very low number of gonocyte colonies observed by Day 7 during short-term culture (Fig. 3e) might be due to their loss through routine medium changes, which apparently eliminated the floating and loosely adherent gonocytes. Therefore, this experiment was designed to examine the medium removed from the plates. The results showed that indeed high numbers of round cells were present in the medium removed routinely every other day. The number of these cells was greatest in the medium removed on Day 2 (6.81 ± 0.63 × 10^4^), compared with Days 4 and 6 (1.02 ± 0.09 × 10^4^ and 0.4 ± 0.07 × 10^4^ cells/well, *P* < 0.05, *n* = 5 replications, Fig. 4). On the other hand, if the medium was not changed, greater numbers of floating gonocytes remained in the medium examined on Days 4 and 6 (4.2 ± 0.10 × 10^4^ and 1.92 ± 0.05 × 10^4^ cells/well, respectively), compared with the numbers obtained on the same days when the medium was changed every other day (*P* < 0.05, *n =* 5 replications, Fig. 4). Based on the morphology, it was presumed that these cells were gonocytes; they were large and round in shape with prominent nuclei (large nucleus to cytoplasm ratio). The viability of these round cells remained high in all examined days (~85–97%, data not shown). Using immunostaining, these round cells were later confirmed to be gonocytes (Fig. 1g–i).

### Exp. 6: Effect of medium changing regimens on attached gonocytes

This experiment was designed to assess the effects of different medium changing regimens on the number and viability of attached gonocytes. The cells were cultured using the selected culture conditions (3.0 × 10^5^ testis cells/well containing ~40% gonocytes, DMEM^++^, 37 °C) for 1 wk, and medium changing regimens were assigned to groups of plates as follows: 1) to serve as a control group, the cells were seeded with 3 mL of medium, and the medium was changed routinely, every 2 d (routine group, 3 changes in 1 wk); 2) the cells were seeded with 2 mL of medium, followed by the addition of another 1 mL after 24 h, and then every 2 d 1 mL of medium was removed from the well and replaced with 1 mL of fresh medium (modified group); or 3) the cells were seeded with 3 mL of medium but the medium was not changed (unchanged group). On Days 2, 4, 6, and 7, the cultured cells were observed under an inverted microscope and the number of gonocyte colonies in four randomly selected fields at 400× was recorded and cells were collected for evaluation of viability (*n* = 5 replications per group).

**Fig. 5:**
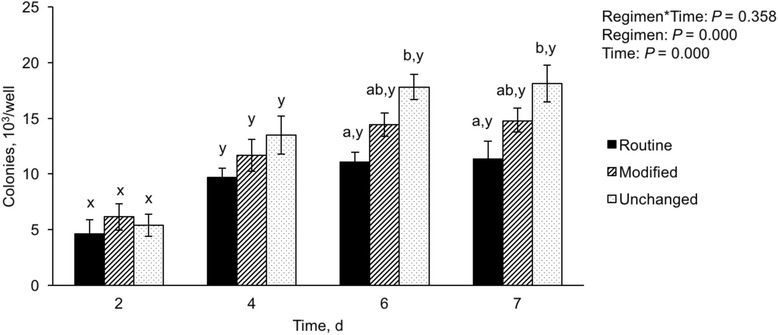
Number of gonocytes or gonocyte colonies observed after applying different medium changing regimens. The cultured cells underwent one of the following medium changing regimens: 1) Seeded with 3 mL of medium and the medium was routinely changed every 2 d (Routine); 2) Seeded with 2 mL of medium, followed by the addition of another 1 mL after 24 h, and then every 2 d, 1 mL of medium was removed from the well and replaced with 1 mL of fresh medium (Modified); 3) Seeded with 3 mL of medium but the medium was not changed for 7 d (Unchanged). The data are mean ± SEM. ^ab^ Data with different superscripts within each day differ significantly (*P* < 0.05). ^xy^ Data with different superscripts within each treatment over time differ significantly (*P* < 0.05). *n* = 5 replications

As shown in Fig. 5, alterations in medium changing regimens affected the number of attached gonocytes or gonocyte colonies present on the somatic-cell monolayers. Overall, the number of gonocyte colonies increased (*P* < 0.05) from Day 2 to Day 4 of culture in all medium changing regimens. However, among the medium changing regimens, the unchanged group had greater (*P* < 0.05) number of gonocyte colonies on Days 6 and 7 compared with the routine medium changing group (control).

**Fig. 6:**
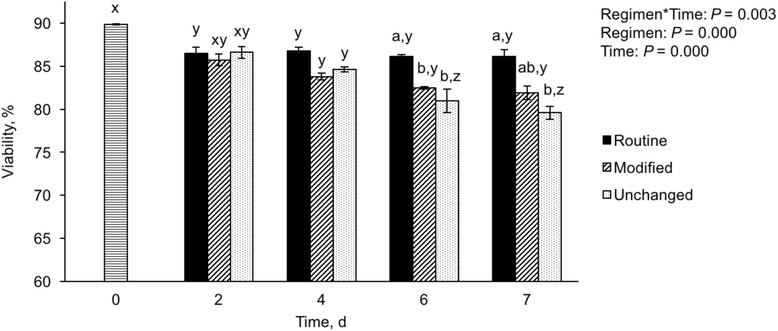
Viability of cells at seeding and after applying different medium changing regimens. The cultured cells underwent one of the following medium changing regimens: 1) Seeded with 3 mL of medium and the medium was routinely changed every 2 d (Routine); 2) Seeded with 2 mL of medium, followed by the addition of another 1 mL after 24 h, and then every 2 d, 1 mL of medium was removed from the well and replaced with 1 mL of fresh medium (Modified); 3) Seeded with 3 mL of medium but the medium was not changed for 7 d (Unchanged). Viability on Day 0 represents the initial viability at seeding. The data are mean ± SEM. ^ab^ Data with different superscripts within each day differ significantly (*P* < 0.05). ^xyz^ Data with different superscripts within each treatment over time differ significantly (*P* < 0.05). *n* = 5 replications

The viability of cells in different medium changing regimens is shown in Fig. 6. Compared with the cell viability at seeding (~90%), there was a reduction in viability of cells at Days 4, 6, and 7 in all groups (*P* < 0.05). The cell viability was reduced further in the unchanged group on Days 6 and 7 (*P* < 0.05). Between the regimens, the viability of cells in the unchanged groups was lower (*P* < 0.05) than the routine group on Days 6 and 7 of culture.

## Discussion

In the neonatal testis, gonocytes represent MGSCs, which have great potential applications in male reproduction research (reviewed in [31, 32]). Various methods have been established to culture MGSCs to facilitate their study and manipulation or to harness their potential for downstream applications such as genetic conservation, transgenesis, or infertility treatments [4, 5, 33–35]. However, depending on the donor species, age, or developmental status, optimized culturing or enrichment of MGSCs can be challenging [29, 36].

Cell isolation is a critical step in preparing testis cells for culture. The number and proportion of the resultant populations of cells also vary greatly depending on the methods used to isolate the cells. As a first step in the present study, we performed a side-by-side comparison of the total testis cell yield and gonocyte proportions from three protocols [29, 30]. Although as expected the enrichment process resulted in highly purified populations of gonocytes (~86%), it also led to losing up to 90% of the initial number of testis cells. These findings provide a basis upon which an appropriate approach could be adopted for gonocyte isolation/enrichment in order to meet the objectives and needs of specific experiments. For example, the highly enriched populations of gonocytes could be used in studies that focus mainly on gonocytes per se, while the heterogeneous populations of testis cells obtained immediately after enzymatic digestion may be more suitable for longer term culture or for the study of interactions between gonocytes and somatic cells.

One of the advantages of using neonatal testes as the source of germ cells is that gonocytes are the only type of germ cells present during the early postnatal stage. Gonocytes possess a distinct morphology and a unique pattern of distribution in the seminiferous cords of neonatal testes, which facilitate their identification in situ [18, 21]. Once the cells are enzymatically dissociated; however, accurate identification of gonocytes requires immunocytochemistry. There are several biomarkers that could be used for identification of gonocytes including germ cell markers (e.g.*,* VASA and DAZL, [37, 38]) and stem cell markers (e.g.*,* Nanog, SSE-A1, and OCT4, [18, 20, 39, 40]). Two of the most commonly used markers for neonatal porcine gonocytes are DBA [18, 19, 41] and ubiquitin C-terminal hydrolase L1 (UCH-L1; previously known as PGP9.5) [41, 42]. DBA has a specific affinity to bind to α-D-N-acetyl-galactosamine, which is localized on the surface of porcine gonocytes (i.e.*,* primitive germ cells) [18], while UCH-L1 can identify both primitive and more advanced germ cells.

In the present study, identification of gonocytes was initially hampered by autofluorescence, which was partly overcome by using a quenching protocol [19]; however, some autofluorescence remained. Autofluorescence in the tissue/cells can be due to endogenous sources (e.g., lipofuscin or lipofuscin-like pigments), or acquired during processing (e.g.*,* certain fixatives). In any case, autofluorescence can interfere with specific signals by immunostaining and lead to false positive results. In the present study, the autofluorescence was eliminated through modification of the quenching protocol [19]. The use of Sudan black B (SBB) has been reported to be efficient in masking autofluorescence of lipofuscins, triglycerides, and lipoproteins origin [43, 44] and is a major component of the quenching protocol for testis cells [19]. In this study, as a first step in modifying the original quenching protocol, the length of incubation with SBB was extended, which led to further reduction of the autofluorescence of testis cells, but not to its complete elimination. We then speculated that the remaining autofluorescence might have a fixative origin. The picric acid component of Bouin’s solution used for fixing testis tissue has also been cited as a potential cause of autofluorescence [45, 46]. Therefore, we applied further control measures to refine the use of fixatives in the quenching protocol. We found that inclusion of three thorough and gentle rinses with 70% alcohol after the Bouin’s fixation completely eliminated the autofluorescence problem in Bouin’s fixed testis cell samples. As an alternative, we also found that to avoid the autofluorescence caused by Bouin’s, cell smears or cells grown on coverslips can be fixed using methanol at −20 °C for 10 min.

Cell seeding density influences the performance of cultured cells, presumably by affecting the contact and interactions among cells [47]. Optimization of the cell seeding density prior to culturing cells ensures normal cell growth and allows estimation of the time to reach certain confluency levels. In the present study, the cell seeding density of 3.0 × 10^5^/well (i.e., in ~10 cm^2^) was found to be more practical because it allowed sampling within 1 wk. without a need for passaging the cells.

Temperature may play an important role in the efficiency of testis cell culture, but the effect of incubation temperature on proliferation of neonatal porcine testis cells has not been well studied. In Exp. 2, testis cells were cultured at either 35 or 37 °C to mimic the testis temperature in situ, which is normally lower than the core body temperature (range 38–40 °C in pigs [48, 49]). Testis cells cultured at either 35 or 37 °C proliferated and reached a 90% confluency within 1 wk.; however, those cultured at 35 °C became 90% confluent later (5.8 vs. 6.4 d, respectively, *P* < 0.05). Earlier studies using human or mouse testis cells concluded that DNA synthesis and steroidogenesis were increased when cells were cultured at 31/32 °C, compared with 37 °C, which is the core body temperature in these species [50, 51]. Although these latter studies also point to the beneficial effects of culturing testis cells at lower than core body temperature, they each examined only one such temperature, making it difficult to compare the results. Results from the present study suggest that further studies will be required to determine the optimal temperature for culturing testis cells for a given species.

The use of poly-L-lysine-coated coverslips is recommended when sampling cultured testis cells. Opposing ionic charges among the cells (polyanionic) and poly-L-lysine-coated coverslip surface (polycationic) enhance efficient attachment of cells onto the coverslips. In the present study, we showed that 1) the presence of poly-L-lysine-coated coverslips had no adverse effects on the growth of cultured testis cells; 2) cells did not grow on non-coated coverslips; and 3) there was no difference in cell viability between the cells growing on coverslips and the peripheral areas of the well. Therefore, while coverslips placed at the bottom of round plate wells provide a convenient method of sampling for analyses such as immunocytochemistry, the cells at the peripheral areas could also be used for other evaluations such as monitoring of the cell viability or use in other assays. Using coverslips also offers the advantage of allowing multiple samplings from the culture dish with minimal disturbance to other cells in the same dish/plate.

In vivo proliferation of gonocytes is influenced by their contact or interaction with the basement membrane of the seminiferous tubule [52]. It has also been suggested that gonocytes require the presence of feeder cells in order to settle and form colonies in vitro [26, 53–56]. In the present study, testis somatic cells formed a monolayer, which provided a platform for subsequent gonocyte attachments. It also appeared that the survival and propagation of gonocytes depended on the proper ratio of gonocytes to somatic cells. This was exemplified in Experiment 1, where we used highly enriched gonocytes (~86%) for seeding and observed no or slow cell proliferation, even when the enriched gonocytes were used at the same seeding density (3.0 × 10^5^/well) as heterogeneous populations of testis cells. This is in agreement with a previous study by van Dissel Emiliani et al. [57] showing that when gonocytes were cultured alone, they failed to adhere to the culture plate and instead formed gonocyte aggregates, which were subsequently lost.

In fetal and neonatal testes, gonocytes are larger than most testis cells and one would expect that among testis cells in the suspension, gonocytes would be one of the first cells to settle. This was indeed the case for mouse fetal gonocytes showing the highest sedimentation velocity of all testis cells [27]. In the present study, however, gonocytes settled much later than other testis cells, and could be seen floating even after several days of culture. Goel et al. [58] examined the population of floating cells after 4 h of culturing neonatal pig testis cells and concluded that they were mostly viable (~95%) and composed of mostly germ cells (91%). In the present study, although the number of floating gonocytes decreased over time, their viability remained high (78–86%) up to 6 d after culture. The extended presence of floating gonocytes may be an advantageous characteristic if they are collected to be used for gonocyte enrichment; however, it can also be disadvantageous and lead to the loss of gonocytes during medium changes, if done without due care.

Changing of medium is important to furnish nutrients and remove waste from a culture system; however, the frequency of medium changing may affect culture conditions. Most studies on testis cell culture use a daily, every-other-day, or twice-weekly approach to medium changing [18, 42, 57, 59, 60]. In the present study, routine changing of medium (every 2 d) reduced the number of attached gonocytes, prompting us to design Experiment 6 for optimizing the medium changing regimen to increase the number of gonocyte colonies. Given our observations of the floating gonocytes, we compared gonocyte colony formation following the routine medium changing regimen (every 2 d) with the group in which the medium was not changed for 1 wk., or with the modified medium changing regimen, where a smaller volume of medium was exchanged at a time. Compared with Day 2 (~5.0 × 10^3^), the number of gonocytes and gonocyte colonies increased in all regimens in subsequent days (Days 4, 6, and 7; Fig. 5). The increased number of gonocyte colonies on Day 4 in all groups (ranging from ~10.0 × 10^3^ to ~13.5 × 10^3^, Fig. 5) could be taken as evidence of gonocyte proliferation in these early days of culture. This conclusion is especially based on the plates in the routine medium changing regimen which had undergone a complete medium change. As a result, this group of plates presumably should have lost all or most of its floating gonocytes, yet there was a 2-fold increase in the number of gonocyte colonies. On the other hand, the number of gonocyte colonies in subsequent days (6 and 7) were ~50% greater in the unchanged group, compared with the routine medium changing regimen (~18.0 × 10^3^ vs. ~12 × 10^3^, respectively, Fig. 5). This can be seen as evidence of continued settlement of gonocytes. The number of gonocyte colonies also increased over time in all groups; this increase was ~3.3 fold for the unchanged medium group (from ~5.4 × 10^3^ on Day 2 to ~18.0 × 10^3^ on Day 7, Fig. 5). This fold-increase in the total number of gonocyte colonies does not take into account that the number of gonocytes within each colony also increased over time (Fig. 3d). Therefore, these results collectively indicate that the optimized culture conditions developed in the present study improved gonocyte colony formation.

In the present study, we systematically examined the effects of several culture conditions on testis cells of the neonatal pig, as a non-rodent animal model. After each experiment, we built upon the newly obtained results and designed subsequent experiments to make successive advances in the survival, propagation, and colony formation of gonocytes. Our results are expected to facilitate further studies aiming at, for instance, investigation of specialized media for large-scale propagation of gonocytes, as a source of MGSCs. Applications for MGSCs range from basic to applied research, including germ cell transplantation studies in farm animals for the purpose of animal transgenesis, genetic conservation, or infertility treatment models [4, 5, 33–35]. Germ cell transplantation can also be used as a functional assay to assess the relative abundance or confirm the developmental potential of MGSCs within a given population of testis cells, including cultured gonocytes (reviewed in [31]).

## Conclusions

In conclusion, the optimized culture conditions developed in the present study included seeding neonatal porcine testis cells at 3.0 × 10^4^ testis cells/cm^2^ containing ~40% gonocytes in DMEM^++^ at 37 °C, and without changing the medium in the first week. These results can be used to improve in vitro maintenance, proliferation, and formation of gonocyte colonies and thereby as a basis for the development of more specialized culture systems for MGSCs.

## Abbreviations

ANOVA: Analysis of variance
DAPI: 4′,6-diamidino-2-phenylindole
DAZL: Deleted in azoospermia like
DBA: *Dolichos biflorus* agglutinin
DMEM: Dulbecco’s modified Eagle medium;
DMEM^++^: DMEM supplemented with 10% fetal bovine serum and 1% antibiotics
DPBS: Dulbecco’s phosphate-buffered saline
ECM: Extracellular matrix
FBS: Fetal bovine Serum
H&E: Hematoxylin and Eosin
HBSS: Hank’s balanced salt solution
HCl: Hydrochloric acid
HSD: Honest significant difference
KHCO_3_: Potassium bicarbonate
MGSCs: Male germline stem cells;
NA_2_EDTA: Disodium ethylenediaminetetraacetate
NANOG: NANOG homeobox
NH_4_Cl: Ammonium chloride
OCT4: Octamer-binding transcription factor 4
PGCs: Primordial germ cells
PGP9.5: Protein gene product 9.5
SEM: Standard error of mean
SPSS: Statistical package for the social sciences
SSCs: Spermatogonial stem cells
UCH-L1: Ubiquitin carboxyl-terminal hydrolase L1
VASA: Dead-box helicase 4
